# Trust in healthcare and perceived discrimination in Sweden: a fixed effects analysis of individual heterogeneity and discriminatory accuracy

**DOI:** 10.3389/fpubh.2025.1557921

**Published:** 2025-06-11

**Authors:** Maria Wemrell, Mariam Hassan, Raquel Perez-Vicente, Martin Lindström, Johan Öberg, Juan Merlo

**Affiliations:** ^1^Department of Social Work, Faculty of Social Sciences, Linnaeus University, Växjö, Sweden; ^2^Unit for Social Epidemiology, Faculty of Medicine, Lund University, Malmö, Sweden; ^3^Department of Health and Medical Care Management, Region Skåne Corporate Office, Malmö, Sweden; ^4^Department of Clinical Sciences Malmö, Section for Family Medicine and Community Medicine, Lund University, Malmö, Sweden; ^5^Centre for Primary Health Care Research, Region Skåne, Malmö, Sweden; ^6^Social Medicine and Health Policy, Faculty of Medicine, Lund University, Malmö, Sweden

**Keywords:** trust in healthcare, discrimination, health inequities, AIHDA, intersectionality, Sweden

## Abstract

**Introduction:**

Trust in healthcare is central to the delivery of care and unequally distributed between groups in society. Experiences of perceived discrimination have been associated with lack of such trust. Although the importance of trust in healthcare has been highlighted in recent years, studies in this area are relatively scarce.

**Materials and methods:**

We investigated the risk of low trust in healthcare in Sweden, using data from 11 consecutive National Public Health Surveys conducted in 2004–2014 (*n* = 83,135). Applying an analysis of individual heterogeneity and discriminatory accuracy (AIHDA), we investigated the risk of low trust in healthcare across intersectional strata defined by experiences of perceived discrimination as well as sex/gender, educational level, migration status and age. We calculated strata-specific prevalences and prevalence ratios (PR) with 95% confidence intervals (CI). The area under the receiver operating characteristic curve (AUC) was computed to evaluate the discriminatory accuracy (DA) of the intersectional strata.

**Results:**

The overall prevalence of low trust in healthcare was 25.9%. While low trust was more common among individuals born abroad, with low education and of younger age, discrimination increased the risk of low trust in healthcare over and above the sociodemographic characteristics. The strata with the highest risk of low trust were foreign-born men aged 55–64 years with low income who had experienced discrimination (PR 3.13 [95% CI 2.49–3.95]) and foreign-born women aged 25–34 years with high education who had experienced discrimination (PR 3.05 [95% CI 2.50–3.73]). The DA of the intersectional strata was small (AUC = 0.64), indicating large overlaps between and heterogeneities within strata.

**Conclusion:**

As experiences of discrimination, in healthcare and elsewhere, are associated with lack of trust in healthcare, it is incumbent on healthcare professionals to maintain trustworthiness by mitigating discriminatory practices including through striving toward patient-centered communication and care. Such efforts should be universal, although proportionally tailored to mitigate discrimination against patients with a migration background.

## Introduction

Trust in healthcare is central to the delivery of care and to the patient-provider relationship. It can affect people’s willingness to seek care, reveal health-related information, participate in screening or testing, receive and adhere to treatment, follow public health recommendations and participate in research (1–4). Trust in healthcare has also been associated with better health outcomes, including self-rated and mental health, higher quality of life and stronger patient satisfaction (2, 5–7). As it is typically not equally distributed between population groups, lack of trust in healthcare can contribute toward and exacerbate health inequities (3, 8–10).

The issue of trust in healthcare has become intensely actualized in recent years, not least in association with the COVID-19 pandemic (4, 11), as changes in public trust in healthcare have been referred to in terms of an erosion or a crisis (12, 13). Research on trust in healthcare is nonetheless relatively scarce [e.g., (9)], as Gille, Smith and Mays (12) indicate a clear imbalance between the centrality of trust for the functioning of healthcare and the low priority awarded to it in research.

Further research has been called for on the antecedents of lack of trust in healthcare (3), on how such trust is distributed between sociodemographic groups (9), how social and health inequalities associated with low trust can be addressed (4), and what behaviors or forms of communication may mitigate or aggravate lack of trust (4). Regarding the latter, studies have shown correlations between distrust in healthcare and previous experiences of discrimination (11, 14–17), i.e., differential and negative treatment of individuals due to being categorized as belonging in a particular demographic group [cf. (18)].

### Aspects and determinants of trust in healthcare

While the lack of a clear definition of trust in healthcare has been noted (12, 19), conceptualizations of trust commonly stress the optimistic acceptance of a vulnerable situation in which the patient or care receiver believes that the healthcare provider will care for the patient’s best interests (1, 9).

Trust in healthcare has been seen to involve trust in the healthcare provider’s competence or expertise and in their motives, i.e., their good will or values aligning with one’s own (15, 20). Hall et al. (1) distinguish between five dimensions of trust, namely overall or global trust alongside trust in the healthcare provider’s fidelity (i.e., pursing the patient’s rather than any other competing interest), competence, honesty and confidentiality. Similarly, Griffiths et al. (19) relate medical trust to the components of healthcare competence, benevolence, integrity, predictability and quality assurance. Trust in healthcare can furthermore be separated into vertical trust in healthcare institutions and horizontal trust, or in the presence of a power gradient vertical trust, in individual healthcare professionals (2). Some have emphasized public or social dimensions of trust in healthcare, as being tied to confidence not only in the safety and efficacy of medical interventions, or in healthcare professionals, but also in the healthcare system, the research community, governmental regulatory or public health agencies, the mass media and society at large (12, 13, 15, 21). Moreover, distinctions can be made between trusting attitudes and trusting behaviors (1, 22) or between epistemic trust in the provided information and recommendation trust expressed through acting on that information (21, 23). Researchers have also distinguished between the evaluation of public or patient trust in healthcare and of the trustworthiness of healthcare, as trust is not necessarily always well founded but can also be misguided or blind (1). This is while lack of trust can be seen as a reasonable and legitimate response to previous negative experiences (19). Rather than categorically trusting or distrusting healthcare, patients can trust healthcare on some issues and not on others, and trust can change based on new information or experiences (19, 22). Due to the importance of building and maintaining trust, Gill, Smith and Mays (24) argue that measures of trust could be used as quality indicators of healthcare systems.

Having found a lower mortality rate among those with moderate trust in the healthcare system, compared to those with high trust, in the context of long waiting times for treatment, Lindström and Pirouzifard (25) conclude that strong trust may be less advantageous for patients through increasing the likelihood of accepting information and waiting times without asking questions or seeking alternatives. Other research shows correlations between trust in healthcare and positive self-rated health outcomes (7). A study using data from the same National Survey of Public Health in Sweden as the current one, although only from the year 2006 (6), showed that very low trust in healthcare was associated with an increased risk of psychological distress.

Lack of trust in healthcare is, as noted, typically not equally distributed across populations. In countries including Sweden, where a regional study found that 31.5% of the population did not strongly trust the health system (9), lower levels of such trust have been identified among racialized groups (3, 8, 9). Low trust in healthcare has also been found in groups with lower income or education (9, 10), although study results diverge results on this point (9, 26). Associations vary with regard to gender, with men reporting lower trust in some populations (27) and women in others (9). More consistently, lower trust has been found in younger age groups (9, 26).

Regarding reasons behind changing or faltering trust in healthcare in recent decades, many discussions have focused on the use of social media and the internet at large (11, 22). Lay online communication about health has often been associated with contestation of expert knowledge and the spread of misinformation (28, 29). The latter has been most intensely discussed in relation to vaccine hesitancy (30, 31), but also in connection with other health issues [e.g., (32, 33)]. While the contemporary online information landscape is of obvious and crucial relevance for communication about and trust in healthcare today, previous research has also pointed to different aspects of healthcare itself as impacting trust. Continuity in care (34), waiting times (35), concerns regarding profit motives in healthcare (13, 15, 22) and the expertise (35–37) and interpersonal skills (1, 35, 37–39) of healthcare providers have thus been shown to affect such trust.

Personal experiences or historical examples of social inequality and discrimination are other factors noted to be associated with lack of trust in healthcare (11, 14–17). Discrimination has been tied to a lack of trust in healthcare when having occurred in a healthcare setting or elsewhere (3, 16), on an interpersonal or a structural level (16). Trust in healthcare has been shown to be affected not only by personal experiences of discrimination, but also by hearing about discrimination against others (3). Direct or vicarious experiences leading to a lack of trust in healthcare have included discrimination alongside stigmatization, poor treatment and inadequate communication in healthcare (17). While discrimination and other forms of differential treatment can be difficult to disentangle (40), measures of personal discrimination (18, 41) have included being treated with less courtesy or respect than other people, receiving poorer services than others or not being listened to. In the contemporary setting, experiences of discrimination have been tied to a lack of trust in healthcare overall as well as to lower acceptance of COVID-19 vaccination (42–44) and other recommended protective behaviors in the context of the pandemic (16).

A large share of studies on links between discrimination and trust in healthcare have focused on racialization (17, 45). Notably, Armstrong et al. (46) found that lower levels of trust in healthcare reported by African Americans, compared to white people, could be explained by experiences of racial discrimination. Discrimination based on other grounds, such as gender, sexual or gender minority status (19, 47), disability (48) or obesity (49), are also likely to have negative effects on trust in healthcare (50, 51). Relatedly, without necessarily specifying the attributed grounds for the treatment, studies conducted in a range of healthcare contexts have linked experiences of not being taken seriously by medical professionals to a lessening of trust in healthcare (22, 32, 35, 36, 38, 52, 53).

In Sweden, lower levels of trust in healthcare have been found among racialized groups (9, 54), and perceived discrimination has been tied to a lack of trust in healthcare among refugees (8). This is while 10% of reported cases of discrimination experienced by individuals with migration backgrounds in this country occurred in healthcare (55). Perceived discrimination has also been associated with refraining from seeking healthcare (56, 57). A lack of trust in healthcare has furthermore been found in more, rather than less, socioeconomically privileged groups in Sweden, as elsewhere (9, 26), as the distribution of such trust may be complex and changing [cf. (26)].

### An intersectional perspective on trust in healthcare

While differences, including complex or potentially changing ones, have thus been found between groups defined by sociodemographic categorizations or experiences of discrimination, research is still scarce, not least in a Swedish context (9). In response to calls for integrating an intersectional perspective in quantitative population health research (58–60), i.e., conceptualizing and analyzing multiple dimensions of privilege and disadvantage in society as interlocking rather than as separate (61), an intersectional perspective can add valuable knowledge about the distribution of low trust in healthcare between population groups. Relatedly, studies of health inequalities including those pertaining to trust in healthcare may oversimplify differences by only assessing average differences between groups, thus disregarding heterogeneities in and overlaps between groups, potentially leading to unnecessary stigmatization of “high–risk” individuals or groups (62, 63). In response to this, assessments of discriminatory accuracy (DA), i.e., the capacity of the groups under study to accurately classify individuals by the outcome of interest (58, 62, 64), can complement such study.

### Aim

This study aims to investigate any associations between lack of trust in healthcare and experiences of perceived discrimination, as well as sociodemographic characteristics, in a nationally representative population sample from Sweden. Applying an analysis of individual heterogeneity and discriminatory accuracy (AIHDA), using an intersectional approach (58, 64, 65), we will examine how trust in healthcare is distributed between groups defined by experiences of perceived discrimination as well as migration background, level of education, sex/gender and age.

## Materials and methods

### Study population and data collection

In this cross-sectional study, we use data from 11 consecutive National Public Health Surveys (NPHS) conducted in Sweden by the Public Health Agency (PHA) (66), in collaboration with Statistics Sweden and the Swedish Association of Local Authorities and Regions, during 2004–2014. The questionnaires included questions about health, lifestyle and living conditions and were distributed annually from 2004 to 2014, online and via post, to a randomized selected population sample encompassing 20,000 individuals aged 16–84 years. The response rates range from 60.8% in 2004 to 48.1% in 2014 (66). The NPHS database provided to us includes sociodemographic data acquired through record-linkage with population registers managed by Statistics Sweden. In the analyses, the data were weighted and expanded using survey weights provided by Statistics Sweden. For more information on the survey and data, see the NPHS reports (67).

**Figure 1:**
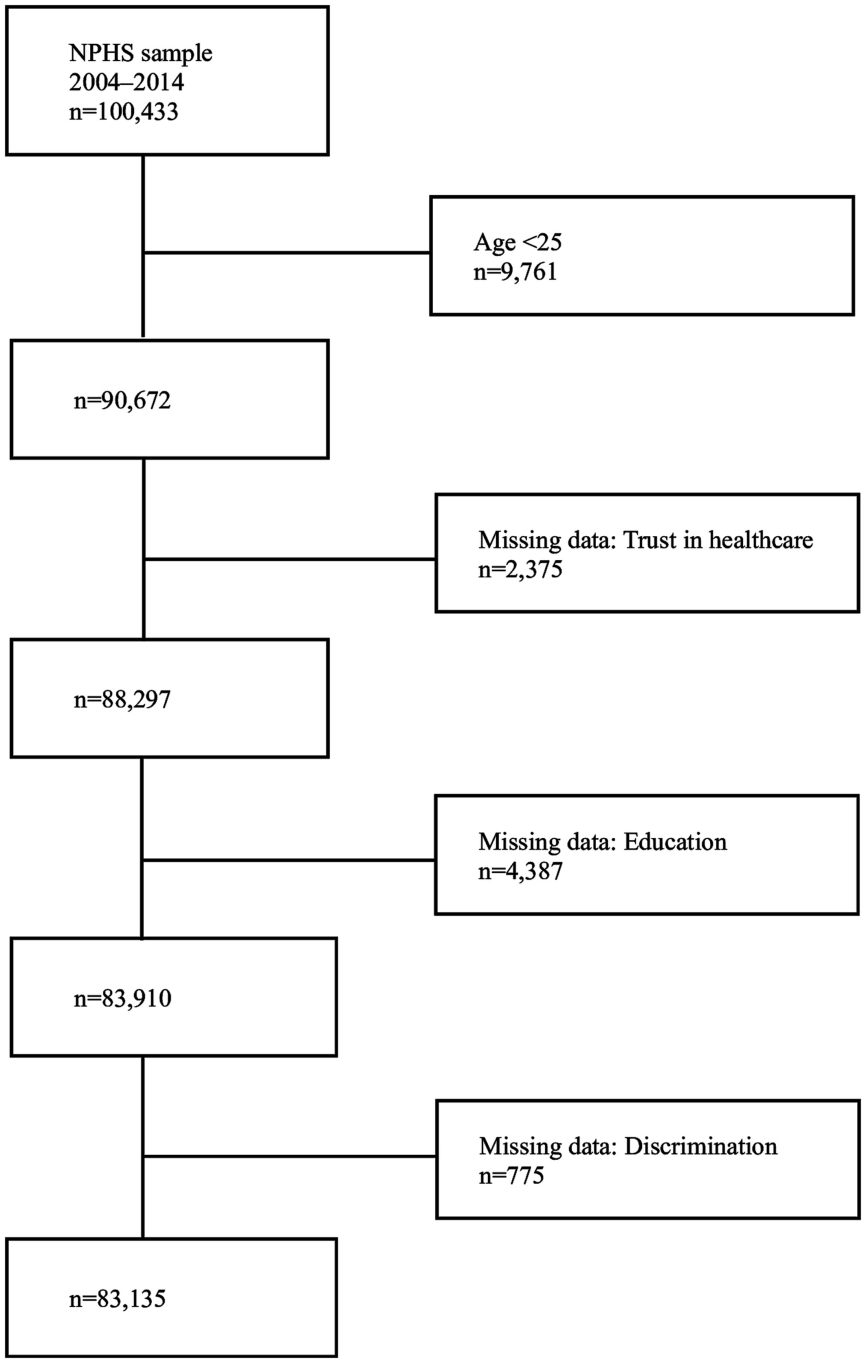
Study sample, with inclusion/exclusion criteria and missing data.

Our sample consists of pooled data from the survey years 2004–2014, encompassing 103,433 respondents. The question about trust in healthcare, which serves as our outcome variable, was removed from 2015 and onwards, which is why we do not include more recent surveys. Participants younger than 25 years (16–24 years) were excluded (*n* = 9,761), as we use education as an indicator of socioeconomic position and younger individuals may not have had sufficient time to attain a higher education. Missing data on trust in healthcare (*n* = 2,375), perceived discrimination (*n* = 775) and educational attainment (*n* = 4,387) were also excluded. Thus, 83,135 individuals, 91.7% of the original sample (from age 25 years), are included in the study (Figure 1).

The study was approved by the Swedish Ethical Review Authorities (2019–01793) and by the PHA’s Ethical Council. NPHS respondents gave informed consent prior to participation.

### Assessment of variables

Our outcome variable is based on the survey question *“How much trust do you have in healthcare,”* posed in conjunction with questions about trust in other societal institutions and enabling responses on a five-point scale. These were dichotomized into *high* vs. *low trust* (*very strong*; *fairly strong*; *have no opinion* vs. *not that strong*; *not at all*).

Perceived discrimination was assessed through the survey question *“During the last three months, have you been treated in a way that made you feel humiliated?,”* the response options being *yes, sometimes*; *yes, several times;* and *no*. Those responding yes were asked about the perceived grounds for this treatment, with response options including *ethnicity*, *sex/gender*, *sexual orientation*, *age*, *disability*, *religion*, *gender identity*, *appearance*, *sexual identity*, *skin color*, *other*, and *do not know*. These options have varied across the survey years, except for *ethnicity*, *sex/gender*, *sexual orientation*, *age*, *disability*, *religion*, *other*, and *do not know*. We therefore used the just noted grounds to construct our variable, in which we categorized those responding *other* or *do not know* as non-discriminated. The variable was dichotomized, categorizing respondents as having experienced perceived discrimination or not (*yes* vs. *no*), based on any of the noted grounds. We use the terms discriminated and discrimination synonymously with the perceived phenomena.

Information on *sex/gender*, obtained from the population register, was coded as *female* vs. *male*. *Age* was classified into four groups (*25–34*; *35–54*; *55–64*; *65–84* years). *Education* was dichotomized as *high* vs. *low*, with secondary schooling (12 years) or less being considered low and any tertiary studies as high. *Migration status* was dichotomized as *native-born* vs. *foreign-born,* with native-born respondents being born in Sweden and foreign-born ones in any other country.

We constructed an intersectional or multicategorical variable by combining the categories of discrimination, sex/gender, age, migration, and education into 64 strata (2 × 2 × 4 × 2 × 2 = 64). The reference stratum used in the analysis was native men aged 35–54 years with a high educational level who had not experienced discrimination. This stratum was assumed to occupy the position of the highest structural privilege and therefore have the lowest risk of reporting low trust in healthcare.

### Statistical analyses

We applied an analysis of individual heterogeneity and discriminatory accuracy (AIHDA) using an intersectional approach, as described in detail elsewhere (58, 64). The purposes of the analysis were (i) to map the distribution of low trust in healthcare across the variables including the 64 intersectional strata, and (ii) to assess the discriminatory accuracy (DA) of the statistical models, i.e., the capacity of the variables used to accurately distinguish between individuals with low versus high trust in healthcare.

We calculated the prevalence or absolute risk, with 95% confidence intervals (CI), of low trust in healthcare in the respective categories and strata. We also assessed the absolute risk difference or attributable risk due to discrimination (ARD), with 95% CIs, for each pair of strata differing only on experiences of discrimination. These measures provide information about the risk of low trust in healthcare that could be eliminated through the absence of experiences of discrimination in the respective strata.

We used eight consecutive regression models to measure differences between categories and strata. Since the prevalence of low trust in healthcare was relatively high (25.9%) we used Cox proportional hazard regression with a constant follow-up time equal to 1 to obtain prevalence ratios (PR) with 95% confidence intervals (CI) (68). Model 1 included only survey year. Model 2 added one variable at a time, with Model 2a including age, Model 2b including sex/gender, Model 2c including migration status, Model 2d including education and Model 2e including discrimination. In Model 3 all these variables were entered simultaneously. Model 4 included the same information but using the multicategorical variable.

To assess the DA of the models, we obtained the received operating characteristics (ROC) curve and calculated the area under the curve (AUC) for each model (69). The ROC curve plots the true positive fraction (sensitivity) against the false positive fraction (1-specificity) across thresholds of predicted probability of low trust in healthcare. The larger the AUC (0.5–1), the larger the DA. In accordance with the criteria proposed by Hosmer and Lemeshow (70), we assess the DA as absent or very small (0.50 ≤ AUC ≤ 0.60), small (0.60 < AUC ≤ 0.70), large (0.70 < AUC ≤ 0.80), or very large (AUC > 0.80).

## Results

The prevalence of low trust in healthcare in the pooled survey database was 25.9% (Table 1A). This prevalence remained relatively stable over the study period, although showed a slight u-shape, as it was higher in the first and final years (29.5% in 2004; 28.5% in 2014) and below 25% during 2008–2011. Overall, 12.4% of the respondents reported very strong trust and 60% indicated fairly strong trust. No opinion was reported by 2%. A small group, 2.7%, responded that they had no trust at all, while 23.2% reported that their trust was not that strong. The prevalence of low trust in healthcare decreased with age, being 8.3% higher in the youngest age group than in the oldest one. Low trust was more common among foreign-born individuals (37%) compared to those born in Sweden (23.9%), among those with low (27.8%) compared to high education (22.6%) and, very slightly so, among females (26.5%) compared to males (25.4%). The prevalence of low trust in healthcare was 19% higher among individuals who had experienced discrimination (44.1%), compared to those who had not (25.0%).

**Table 1A:** Description of the population and prevalence of low trust in healthcare, by categories of age, sex/gender, migration status, education and perceived discrimination, using NPHS data from 2004 to 2014.

Categories	Population (*n* = 83,135)	Prevalence of low trust
	n (%)	*p* (95% CI)
	Unweighted data	Weighted data
Trust		
High	62,880 (75.6%)	4,285,230 (74.1%)
Low	20,255 (24.4%)	1,500,664 (25.9%)
Age		
25–34	12,110 (14.6%)	29.0% (28.1–29.9)
35–54	31,451 (37.8%)	27.4% (26.8–27.9)
55–64	18,578 (22.4%)	25.4% (24.8–26.1)
>65	20,996 (25.3%)	20.7% (20.1–21.3)
Sex/gender		
Male	37,919 (45.6%)	25.4% (24.9–25.9)
Female	45,216 (54.4%)	26.5% (26.1–27.0)
Migration status		
Native–born	73,189 (88.0%)	23.9% (23.5–24.2)
Foreign–born	9,946 (12.0%)	37.0% (35.9–38.0)
Education		
Low	50,544 (60.8%)	27.8% (27.3–28.2)
High	32,591 (39.2%)	22.6% (22.1–23.1)
Discrimination		
Yes	3,894 (4.7%)	44.1% (42.4–45.8)
No	79,241 (95.3%)	25.0% (24.7–25.3)

Table 1B shows the prevalence of low trust in healthcare in the 64 intersectional strata. In 12 strata, the share of respondents reporting low trust in healthcare was above 50%. All these strata comprised individuals who had experienced discrimination. Eleven included foreign-born individuals, the exception being native-born men aged 25–34 years with a low education who had experienced discrimination. Eight of the strata comprised men, and seven included individuals with low education. The highest prevalence of low trust was found among foreign-born men with low education, 50–64 years old, who had experienced discrimination (64.2% [95% CI 48.8–77.1]) and foreign-born women aged 25–34 years with high education, who had experienced discrimination (62.4% [95% CI 50.1–73.3]).

**Table 1B:** Prevalence of low trust in healthcare, with 95% confidence intervals, for intersectional strata combining categories of age, sex/gender, migration status, education and experiences of perceived discrimination, using weighted NPHS data from 2004 to 2014.

Age	Sex/gender	Migration status	Education	Discrimination	Low trust; *p* (95% CI)
25–34	Women	Foreign-born	Low	No	37.8 (32.2–43.7)
				Yes	48.2 (34.1–62.5)
			High	No	37.2 (32.5–42.2)
				Yes	62.4 (50.1–73.3)
		Native-born	Low	No	32.4 (30.4–34.5)
				Yes	44.6 (38.3–51.2)
			High	No	21.3 (19.8–22.9)
				Yes	38.6 (34.0–43.4)
	Men	Foreign-born	Low	No	39.3 (33.5–45.4)
				Yes	50.7 (36.9–64.4)
			High	No	36.3 (30.4–42.5)
				Yes	54.4 (34.7–72.8)
		Native-born	Low	No	29.2 (27.3–31.3)
				Yes	53.9 (44.0–63.4)
			High	No	20.3 (18.6–22.2)
				Yes	25.3 (15.7–38.1)
35–54	Women	Foreign-born	Low	No	40.8 (37.8–43.9)
				Yes	52.2 (42.5–61.8)
			High	No	36.9 (33.8–40.2)
				Yes	48.7 (40.1–57.3)
		Native-born	Low	No	27.3 (26.3–28.4)
				Yes	45.7 (40.4–51.1)
			High	No	18.3 (17.3–19.3)
				Yes	29.6 (24.9–34.8)
	Men	Foreign-born	Low	No	38.4 (35.2–41.8)
				Yes	57.1 (46.9–66.7)
			High	No	32.7 (29.1–36.5)
				Yes	56.2 (45.5–66.3)
		Native-born	Low	No	27.2 (26.1–28.3)
				Yes	41.3 (33.8–49.3)
			High	No	20.5 (19.3–21.8)
				Yes	39.9 (30.0–50.7)
55–64	Women	Foreign-born	Low	No	38.7 (34.8–42.7)
				Yes	54.8 (40.4–68.4)
			High	No	29.8 (24.9–35.3)
				Yes	53.9 (38.5–68.7)
		Native-born	Low	No	26.3 (25.0–27.5)
				Yes	39.3 (32.1–47.1)
			High	No	19.2 (17.7–20.7)
				Yes	31.5 (24.3–39.7)
	Men	Foreign-born	Low	No	33.8 (29.7–38.1)
				Yes	64.2 (48.8–77.1)
			High	No	31.1 (25.6–37.2)
				Yes	50.5 (31.9–69.0)
		Native-born	Low	No	24.3 (23.0–25.5)
				Yes	42.6 (34.5–51.2)
			High	No	17.0 (15.5–18.6)
				Yes	28.4 (18.6–40.7)
>65	Women	Foreign-born	Low	No	26.5 (23.4–29.9)
				Yes	49.3 (35.6–63.1)
			High	No	28.5 (22.7–35.2)
				Yes	40.9 (22.7–62.0)
		Native-born	Low	No	21.2 (20.2–22.3)
				Yes	36.6 (29.3–44.6)
			High	No	16.7 (15.1–18.4)
				Yes	38.2 (29.9–47.4)
	Men	Foreign-born	Low	No	24.0 (20.7–27.6)
				Yes	55.8 (37.5–72.6)
			High	No	22.8 (17.3–29.4)
				Yes	25.2 (7.5–58.5)
		Native-born	Low	No	18.9 (17.9–19.9)
				Yes	44.9 (36.4–53.6)
			High	No	14.6 (13.1–16.3)
				Yes	18.5 (10.2–31.1)

In seven strata, the prevalence of low trust was lower than 20%. All of these included native-born individuals. Six comprised people who had not experienced discrimination, the exception being a stratum including native men with high education, aged 65 years or more. Six of the strata included people with high education, four included men and four were comprised of individuals aged 65 years or more. The lowest prevalence was found among native-born men and women with high education, aged 65 years or more, who had not experienced discrimination (14.6% [95% CI 13.1–16.3]; 16.7% [95% CI 15.1–18.4]).

Accordingly, the regression analysis of the intersectional strata (Model 4; Table 2A) showed that in 10 strata the risk of low trust in healthcare was 2.5 to 3.1 times higher than in the reference stratum. Again, all but one of the strata with the highest risk included foreign-born individuals who had experienced discrimination, whereas their sex/gender, age and level of education varied.

**Table 2A:** The 10 strata with the highest and lowest risk of low trust in healthcare, obtained through regression analysis (Model 4) using weighted NHPS data from 2004 to 2014.

Intersectional strata	*p* (95% CI)	PR (95% CI)
10 strata with the highest risk		
Discrimination; Foreign-born; 55–64 yrs.; Men; Low education	64.2 (48.8–77.1)	3.13 (2.49–3.95)
Discrimination; Foreign-born; 25–34 yrs.; Women; High education	62.4 (50.1–73.3)	3.05 (2.50–3.73)
Discrimination; Foreign-born; 35–54 yrs.; Men; Low education	57.1 (46.9–66.7)	2.80 (2.32–3.38)
Discrimination; Foreign-born; >65 yrs.; Men; Low education	55.8 (37.5–72.6)	2.78 (1.99–3.87)
Discrimination; Foreign-born; 35–54 yrs.; Men; High education	56.2 (45.5–66.3)	2.77 (2.27–3.38)
Discrimination; Foreign-born; 25–34 yrs.; Men; High education	54.4 (34.7–72.8)	2.67 (1.85–3.86)
Discrimination; Foreign-born; 55–64 yrs.; Women; Low education	54.8 (40.4–68.4)	2.64 (2.02–3.47)
Discrimination; Foreign-born; 55–64 yrs.; Women; High education	53.9 (38.5–68.7)	2.63 (1.96–3.52)
Discrimination; Native-born; 25–34 yrs.; Men; Low education	53.9 (44.0–63.4)	2.63 (2.18–3.18)
Discrimination; Foreign-born; 35–54 yrs.; Women; Low education	52.2 (42.5–61.8)	2.53 (2.08–3.09)
10 strata with the lowest risk		
No discrimination; Native-born; >65 yrs.; Men; High education	14.6 (13.1–16.3)	0.71 (0.63–0.81)
No discrimination; Native-born; >65 yrs.; Women; High education	16.7 (15.1–18.4)	0.81 (0.72–0.91)
No discrimination; Native-born; 55–64 yrs.; Men; High education	17.0 (15.5–18.6)	0.83 (0.74–0.93)
No discrimination; Native-born; 35–54 yrs.; Women; High education	18.3 (17.3–19.3)	0.89 (0.82–0.97)
Discrimination; Native-born; >65 yrs.; Men; High education	18.5 (10.2–31.1)	0.91 (0.52–1.61)
No discrimination; Native-born; >65 yrs.; Men; Low education	18.9 (17.9–19.9)	0.92 (0.85–1.00)
No discrimination; Native-born; 55–64 yrs.; Women; High education	19.2 (17.7–20.7)	0.93 (0.85–1.03)
No discrimination; Native-born; 25–34 yrs.; Men; High education	20.3 (18.6–22.2)	0.99 (0.89–1.10)
*No discrimination; Native-born; 35–54 yrs; Men; High education*	20.5 (19.3–21.8)	*(Reference)*
No discrimination; Native-born; 25–34 yrs.; Women; High education	21.3 (19.8–22.9)	1.04 (1.95–1.14)

The values are prevalences and prevalence ratios with 95% confidence intervals.

Table 2B shows the ARs of low trust in healthcare in pairs of similar strata differing only on reported discrimination, as well as the ARD attributable to discrimination for each strata pair. Of the 32 strata, 29 showed an ARD ≥ 10, and nine had an ARD ≥ 20. This is while the ARD of eight of the 32 strata were inconclusive. The highest ARD, of 31.81 and 30.43, respectively, were found among foreign-born men with low education, aged 65 + and 55–64. Of the nine strata with an ARD ≥ 20, six included foreign-born individuals and three native-born ones. Five comprised men and five persons with low education.

**Table 2B:** Prevalence or absolute risk (AR) of low trust in healthcare, and absolute risk difference (ARD) attributable to perceived discrimination, with 95% confidence intervals (CI) among pairs of otherwise similar intersectional strata, using weighted NHPH data from 2004 to 2014.

Age	Sex/gender	Migration status	Edu-cation	No discrimination	Discrimination	ARD
25–34	Women	Foreign-born	Low	37.79 (32.20–43.73)	48.17 (34.11–62.52)	10.38 (−5.34–26.09)
			High	37.31 (32.50–42.18)	62.38 (50.09–73.26)	25.17 (12.43–37.91)
		Native-born	Low	32.43 (30.42–34.52)	44.59 (38.28–51.09)	12.16 (5.40–18.92)
			High	21.31 (19.83–22.88)	38.60 (34.01–43.41)	17.29 (12.33–22.25)
	Men	Foreign-born	Low	39.31 (33.50–45.43)	50.68 (36.85–64.41)	11.37 (−3.99–26.74)
			High	36.25 (30.39–42.54)	54.38 (34.71–72.78)	18.13 (−2.81–39.08)
		Native-born	Low	29.24 (27.27–31.28)	53.85 (44.02–63.38)	24.61 (14.60–34.62)
			High	20.34 (18.59–22.21)	25.27 (15.65–38.12)	4.93 (−6.54–16.40)
35–54	Women	Foreign-born	Low	40.84 (37.82–43.93)	52.18 (42.45–61.75)	11.34 (1.10–21.58)
			High	36.92 (33.77–40.17)	48.65 (40.07–57.31)	11.73 (2.45–21.01)
		Native-born	Low	27.33 (26.27–28.40)	45.69 (40.37–51.11)	18.37 (12.87–23.86)
			High	18.29 (17.31–19.31)	29.61 (24.88–34.83)	11.33 (6.24–16.41)
	Men	Foreign-born	Low	38.42 (35.19–41.75)	57.08 (46.88–66.72)	18.67 (8.10–29.24)
			High	32.71 (29.12–36.51)	56.18 (45.49–66.32)	23.47 (12.28–34.67)
		Native-born	Low	27.21 (26.12–28.32)	41.33 (33.78–49.31)	14.13 (6.22–22.03)
			High	20.50 (19.27–21.79)	39.86 (29.97–50.65)	19.36 (8.80–29.92)
55–64	Women	Foreign-born	Low	38.70 (34.82–42.73)	54.79 (40.40–68.42)	16.09 (1.16–31.02)
			High	29.83 (24.91–35.26)	53.94 (38.47–68.68)	24.11 (7.68–40.53)
		Native-born	Low	26.25 (25.02–27.52)	39.32 (32.06–47.09)	13.07 (5.40–20.74)
			High	19.15 (17.71–20.69)	31.50 (24.28–39.73)	12.34 (4.43–20.26)
	Men	Foreign-born	Low	33.77 (29.74–38.05)	64.20 (48.80–77.14)	30.43 (15.32–45.55)
			High	31.09 (25.55–37.22)	50.51 (31.92–68.96)	19.42 (−0.89–39.73)
		Native-born	Low	24.27 (23.04–25.54)	42.63 (34.47–51.22)	18.36 (9.81–26.91)
			High	16.99 (15.47–18.62)	28.35 (18.59–40.69)	11.36 (0.80–22.65)
65–	Women	Foreign-born	Low	26.49 (23.35–29.89)	49.34 (35.63–63.14)	22.84 (8.35–37.34)
			High	28.54 (22.72–35.18)	40.87 (22.68–61.96)	12.33 (−9.32–33.97)
		Native-born	Low	21.24 (20.24–22.28)	36.60 (29.32–44.55)	15.36 (7.62–23.10)
			High	16.70 (15.10–18.43)	38.23 (29.87–47.35)	21.53 (12.55–30.51)
	Men	Foreign-born	Low	23.98 (20.73–27.55)	55.78 (37.49–72.63)	31.81 (13.15–50.46)
			High	22.76 (17.25–29.41)	25.23 (7.49–58.45)	2.47 (−25.14–30.08)
		Native-born	Low	18.87 (17.91–19.88)	44.86 (36.43–53.59)	25.98 (17.26–34.70)
			High	14.62 (13.07–16.31)	18.45 (10.18–31.09)	3.83 (−6.68–14.34)

The DA of the strata was small (Table 3), with AUC = 0.61 (0.60–0.61) in Model 4 which includes the intersectional strata. That is, while substantial average differences can be seen between the strata, the group membership’s ability to classify individuals with low vs. high trust is limited due to large individual heterogeneity.

**Table 3:** Prevalence ratios of low trust in healthcare, with 95% confidence intervals, calculated in eight consecutive regression analyses using weighted NHPH data from 2004 to 2014.

PR (95% CI)							
Age	*Model 1*	*Model 2a*	*Model 2b*	*Model 2c*	*Model 2d*	*Model 2e*	*Model 3*	*Model 4*
25–34		1.40 (1.34–1.46)					1.45 (1.39–1.51)	
35–54		1.33 (1.28–1.37)					1.35 (1.30–1.40)	
55–64		1.23 (1.19–1.28)					1.25 (1.20–1.30)	
>65		*(Reference)*					*(Reference)*	
Sex/gender								
Male			*(Reference)*				*(Reference)*	
Female			1.05 (1.02–1.07)				1.04 (1.02–1.07)	
Migration status								
Native-born				*(Reference)*			*(Reference)*	
Foreign-born				1.55 (1.50–1.60)			1.49 (1.44–1.53)	
Education								
Low					1.22 (1.19–1.26)		1.32 (1.28–1.36)	
High					*(Reference)*		*(Reference)*	
Discrimination								
Yes						1.78 (1.70–1.85)	1.61 (1.54–1.68)	
No						*(Reference)*	*(Reference)*	
Survey year								
2004	*(Reference)*	*(Reference)*	*(Reference)*	*(Reference)*	*(Reference)*	*(Reference)*	*(Reference)*	*(Reference)*
2005	0.94 (0.89–1.00)	0.94 (0.88–0.99)	0.94 (0.89–1.00)	0.94 (0.89–1.00)	0.94 (0.89–1.00)	0.98 (0.93–1.04)	0.99 (0.93–1.05)	0.99 (0.93–1.04)
2006	0.91 (0.85–0.96)	0.91 (0.86–0.96)	0.91 (0.85–0.96)	0.90 (0.85–0.96)	0.91 (0.86–0.97)	0.91 (0.86–0.96)	0.91 (0.86–0.97)	0.91 (0.86–0.97)
2007	0.89 (0.83–0.94)	0.89 (0.83–0.94)	0.89 (0.83–0.94)	0.89 (0.83–0.94)	0.89 (0.84–0.95)	0.89 (0.84–0.95)	0.90 (0.85–0.96)	0.90 (0.84–0.95)
2008	0.79 (0.75–0.84)	0.80 (0.76–0.84)	0.79 (0.75–0.84)	0.79 (0.75–0.83)	0.80 (0.76–0.85)	0.80 (0.76–0.85)	0.81 (0.77–0.85)	0.81 (0.77–0.85)
2009	0.83 (0.78–0.87)	0.83 (0.79–0.87)	0.83 (0.78–0.87)	0.82 (0.77–0.86)	0.84 (0.79–0.88)	0.83 (0.79–0.88)	0.84 (0.80–0.89)	0.84 (0.80–0.89)
2010	0.78 (0.74–0.82)	0.80 (0.76–0.84)	0.78 (0.74–0.82)	0.77 (0.73–0.82)	0.79 (0.75–0.83)	0.79 (0.75–0.83)	0.81 (0.77–0.85)	0.81 (0.77–0.85)
2011	0.82 (0.78–0.86)	0.84 (0.79–0.88)	0.82 (0.78–0.86)	0.81 (0.77–0.85)	0.83 (0.78–0.87)	0.82 (0.78–0.87)	0.85 (0.80–0.89)	0.84 (0.80–0.89)
2012	0.85 (0.81–0.90)	0.87 (0.83–0.92)	0.85 (0.81–0.90)	0.84 (0.80–0.88)	0.86 (0.82–0.91)	0.86 (0.82–0.90)	0.89 (0.84–0.93)	0.88 (0.84–0.93)
2013	0.92 (0.88–0.97)	0.95 (0.90–0.99)	0.92 (0.88–0.97)	0.91 (0.87–0.96)	0.93 (0.89–0.98)	0.92 (0.88–0.97)	0.96 (0.91–1.01)	0.96 (0.91–1.00)
2014	0.97 (0.92–1.02)	0.99 (0.94–1.04)	0.97 (0.92–1.02)	0.95 (0.91–1.00)	0.98 (0.93–1.03)	0.97 (0.93–1.02)	1.01 (0.96–1.06)	1.01 (0.96–1.06)	
DA (AUC)	0.53 (0.53–0.54)	0.56 (0.55–0.56)	0.53 (0.53–0.54)	0.56 (0.56–0.57)	0.55 (0.55–0.56)	0.55 (0.55–0.56)	0.61 (0.60–0.61)	0.61 (0.60–0.61)

## Discussion

This cross-sectional study analyzed the effects of perceived discrimination and sociodemographic variables on trust in healthcare in Sweden, using data from the National Public Health Survey and adopting an AIHDA using an intersectional approach.

Of the survey respondents, 25.9% reported low trust in healthcare. This is similar to the result of a study conducted in northern Sweden, where 31.5% reported low trust in the health system (9). The difference between the two studies is not large, but can be accounted for by the latter excluding those reporting no opinion whereas we included them in the high-trust group, the latter being based on data from 2014 when the prevalence of low trust was at the second highest level in our 2004–2014 data, and the latter being focused on a northern region of Sweden while our sample was nation-wide.

Our finding that trust was lower among foreign-born individuals is also in line with previous research from Sweden and elsewhere, where lower trust was observed among persons with migration background, or with racialized or minority status (3, 8, 9). Other studies have also, like us, found lower trust in younger age groups (9, 26) and among those with lower education, although associations between trust in healthcare and income or educational levels have varied (9, 10, 26). Whereas previous research have observed differing associations with gender (9, 27), we saw no big differences between levels of trust among men and women. Notably, and in line with previous research (3, 15, 17), the strongest association with low trust in healthcare was that of having experienced discrimination.

This association between low trust in healthcare and having experienced discrimination was strengthened in an additional analysis, which distinguished the group reporting no trust at all from those indicating some trust in healthcare. The results of this analysis are available in the supplementary material (S1). Those in the small group reporting no trust at all in healthcare (2.7%) were nearly three times more likely to have experienced discrimination than to have not. Individuals in this group were also more than twice as likely to have a migration background than to be native-born.

The analysis of the intersectional strata, providing a more detailed mapping of the distribution of trust, showed a risk of low trust in healthcare three times higher in some strata compared to the reference group, although a low DA indicated substantial heterogeneities in and overlaps between groups. The strata with the highest prevalence were united by their experiences of discrimination and, in all cases except one, by their migration background, while their gender, age and educational level differed. The stratum comprising individuals without a migration background included men with low education, aged 25–35 years, which invites questions about the nature of the here experienced perceived discrimination. The strata with the lowest risk of low trust in healthcare included individuals born in Sweden who had, in all strata but one, not experienced discrimination. The exception was men aged 65 + with high education, who may have experienced discrimination due to, for example, age, sexual orientation or disability, but who still had strong trust in healthcare.

Moreover, the ARD due to discrimination, i.e., the risk of low trust in healthcare that could be eliminated in the absence of experiences of discrimination in each stratum, was above 10% in 29 of the 32 strata, and above 20% in nine of them.

All these analyses consider discrimination irrespective of where it occurred. However, in 2004–2005, the NPHS posed a question about where or by who the respondent was subjected to humiliating treatment (e.g., *healthcare*, *public employment service*, or *by close relatives*). An additional analysis of this data, the results of which can be found in the supplementary material (S2), showed that of those reporting having experienced discrimination in a healthcare setting, as many as two-thirds (65.6%) indicated low trust in healthcare. This result, which warrants an emphasis, indicates a clear correlation between low trust in healthcare and experiences of perceived discrimination in a healthcare setting.

### Mitigating lack of trust in healthcare through avoiding discrimination

In recent years, lack of trust in healthcare has often been discussed in connection to online lay communication about health-related matters (33), including concerns about the spreading of misinformation (28) in the context of significant societal polarization (71). While such contemporary processes are undoubtedly highly relevant, among different and complex factors affecting trust in healthcare today [e.g., (11)], this study focuses on the link between lack of such trust and experiences of discrimination occurring in healthcare and elsewhere. This link underscores that healthcare professionals and institutions have an important part to play in the building and maintaining of public trust in healthcare, not least among more marginalized populations (14, 42, 72), including through demonstrating or increasing trustworthiness (13, 73) by avoiding discriminatory practices (16, 19).

While discrimination occurring anywhere can affect trust in healthcare (3, 16), healthcare professionals and institutions obviously have limited abilities to respond to discrimination happening elsewhere. They can, however, mitigate discrimination in healthcare settings [cf. (18)].

Whereas further research has been called for on what behaviors and other contributing factors that elicit and sustain trust, how communication establishes, maintains, increases or diminishes trust, and how trust-and relationship-based approaches may help address health inequities (4), existing research (17) indicates that discrimination, alongside bias, stigmatization and inadequate communication, counteract trust in healthcare. Jaiswal (14) points out, for example, that while discrimination and stigma shape lack of trust in healthcare, trust is built through respectful interaction, clear and honest communication and provision of patient choice. Similarly, studies have found that healthcare providers’ interpersonal skills, such as taking patients seriously and treating patients with respect, have been strongly associated with patient trust (35, 38, 39). This has also been indicated in studies of care for racialized minorities, including in Sweden (8), where experiences of being dismissed, receiving inadequate care, and facing racism, have been associated with lack of trust, delay in seeking healthcare and going abroad to seek care (8). Lack of trust in healthcare can also arise from discrimination based on other grounds [e.g., (19, 50, 51, 74)]. Without specifying the grounds for such treatment, and sometimes referring to limitations in patient-centered care rather than discrimination, studies have pointed to experiences of not being taken seriously, or not being listened to or supported, as being tied to a lessening of trust in healthcare in contexts including rare forms of cancer (36), endometriosis (37), chronic pain (53), contraceptive care (32, 52), and use of forms complementary or fringe medicine (22). Accordingly, the importance of patient-provider communication (6, 12), or of relationship-centered (11) or patient-centered care (32, 35), for the building of trust has been repeatedly emphasized. For this purpose, patient-centered communication should be respectful, emphatic, actively listening and involve adequate sharing of information (11, 12, 36, 72).

Our increasingly heterogeneous information environment can be seen to only increase the importance of healthcare providers doing what they can to safeguard patient trust (75). In this context, maintaining patient trust through patient-centered communication can encompass willingness toward open communication and dialog about patient concerns potentially including diverse issues or sources of information (22). This is while experiences of being dismissed or stigmatized by healthcare professionals have been noted among reasons for turning to online support groups (32, 37), and as experiences of discrimination have been shown to affect adherence to counter-authority beliefs regarding COVID-19 (16). Commenting on COVID-19 vaccine uptake, Paul et al. (42) observe that focusing only on aspects of information or misinformation will likely not serve to address mistrust that is largely due to past experiences of discrimination. Relatedly, it has been observed that practices of dismissing or ridiculing those hesitant or skeptical toward public health recommendations seen during the COVID-19 pandemic are likely to have diminished rather than augmented trust in healthcare (21), and researchers (76–78) have argued against practices of online content moderation, deplatforming or censorship of health-related content for reasons including that it will likely deepen lack of trust in healthcare. That is, while it is obviously important to counter false information (11), open and respectful communication and discussion remain highly important for the maintaining of trust in healthcare [e.g., (12)]. In the contemporary setting, efforts to mitigate lack of trust in healthcare should not disregard the importance of avoiding discrimination, including by strengthening patient-centered communication and care, for maintaining such trust.

There is a relative lack of research on interventions or strategies to reduce discrimination in healthcare settings, and on their effects (79, 80). Such interventions have often been educational, aiming to increase self-reflection, humility and awareness of unconscious bias among healthcare providers, and to improve patient-provider interaction (79, 81, 82). In a small educational intervention conducted in medical training in Sweden (83), for example, case studies constructed after interviews about racism with healthcare staff were discussed and reflected on by students. Another suggested strategy is to begin the educational intervention with a survey in the participant group, as a basis for further discussions and in order to customize the training (84). While such educational elements can be highly important, it has been emphasized that these should be combined with interventions on organizational and policy levels, including long-term reflective approaches, supportive systemic structures, and the recruitment and retention of healthcare providers from minority groups (80, 81, 85). It should be noted here that healthcare providers and students also experience discrimination, by patients (86), teachers, co-students and colleagues (87), and that this needs to be acknowledged and addressed as part of efforts to avoid discrimination in healthcare. Accordingly, and in sum, Smith et al. (16) note that due to its impact on trust in healthcare, explicit and implicit discriminatory practices should be acknowledged and actively mitigated both on the individual level of medical professionals and on institutional levels.

This study presents a mapping of population strata with large average differences in trust in healthcare. At the same time, the DA of those strata is low. That is, while the average risk of low trust is higher for example in groups comprising individuals with a migration background, many persons reporting low trust can be found in the numerically larger groups consisting of people born in Sweden. This is in alignment with the preventive paradox outlined by Rose (88). Accordingly, efforts to avoid discrimination in healthcare should be universal, i.e., directed to the whole population, although proportionally targeted (89) to mitigate discrimination against specific strata such as those including patients with a migration background. This study provides information that can aid the tailoring of such efforts.

Finally, our survey data were gathered 10–20 years ago. Since then, the discrimination of foreign-born individuals has likely not decreased in Sweden (55), as immigration policies have become more restrictive and political debates have included a push for reduced entitlement to healthcare for refugees (90, 91).

### Limitations

Alongside its strengths, which include being based on a large national-level data sample, this study has its limitations. Some of these pertain to the variables used. As noted, the concept of trust in healthcare can be conceptualized differently (1, 12, 19) and seen to incorporate various dimensions, such as trust in expertise and motives (20, 37), professionals and institutions (2) or trusting attitudes and behaviors (23). The variable used to measure trust in this study is simplistic, and further research should investigate dimensions of it more closely.

Regarding the measurement of discrimination, perceptions of whether a certain treatment was discriminatory or not may differ between individuals, as perceived or verifiable discrimination and other forms of differential treatment can be hard to disentangle (40). Still, associations between perceived discrimination and lack of trust in healthcare have been repeatedly shown [e.g., (17)]. Furthermore, our variable fails to encompass the full scope of experiences of discrimination, as it includes responses to survey questions about only five grounds for such treatment. As we excluded grounds of discrimination introduced in the NPHS in more recent years, including skin color and gender identity, such response options may have been chosen as the attributed reason for the discriminatory treatment. Thus, the effects of discrimination on lack of trust in healthcare may have been underestimated. Also, only a small additional analysis using data from 2004 to 2005 pertains specifically to experiences of discrimination in healthcare, as that was the only available data permitting this analysis. In addition, we do not know to what extent the experienced discrimination can be understood to result from conscious or unconscious bias, or other factors such as poor communication skills.

Limitations pertain to the variables included in the intersectional strata. Other relevant dimensions, such as sexual orientation, were not included, sex/gender was included only as a binary variable, and the migration status variable (*native-born* vs. *foreign-born*) was very simple (92). A more complex migration status variable, distinguishing between different countries or areas of birth, would have been helpful. This would, however, have strongly increased the number of intersectional strata and many of the strata comprising individuals with a migration background would have been very small. We avoided using intersectional strata containing few or no individuals for the purpose of the statistical analysis, and our variables were based on the data available from the NPHS. Further research on the effects of discrimination on trust in healthcare should examine a more complete selection of grounds for discrimination, while investigating discrimination in healthcare settings and elsewhere.

The survey response rate was limited, at 48.1–60.8%, and decreased over time (66). A non-response analysis of an earlier edition of the NPHS showed that people in younger age groups, born outside Sweden, and with lower education were less likely to respond (93). As this tendency may increase alongside a decreasing response rate, our results may have been affected by survey response selection bias. To address this issue our results were weighted, using weights provided by Statistics Sweden, but this does not eliminate the risk of selection bias.

We used a cross-sectional design, appropriate for the investigation of the prevalence of low trust in healthcare and of correlations between low trust and experienced discrimination as well as sociodemographic variables. This design limits the basis on which the study can be used to draw inferences of causality, however. It could have been conducted using a multilevel MAIHDA approach (58), which was developed for assessing the impacts of intersecting identities or variables, and is rooted in intersectionality theory (60) although it can also be used for other forms of multicategorical analyses [e.g., (94)]. MAIHDA has clear methodological and conceptual advantages (58, 60, 95). Still, the AIHDA fixed effects approach shares many of those advantages, such as providing an intersectional mapping of average risk and assessing DA. In addition, AIHDA is more accessible and therefore suitable for the analysis of public health surveys or reports [cf. (64)].

## Conclusion

As experiences of discrimination in healthcare and elsewhere are associated with lack of trust in healthcare, it is incumbent on healthcare professionals to maintain trustworthiness by mitigating discriminatory practices including through striving toward patient-centered communication and care. Such efforts should be universal, although proportionally tailored to mitigate discrimination against patients with a migration background.

## Data Availability

The data analyzed in this study is subject to the following licenses/restrictions: the data that support the findings of this study are available from the Swedish Public Health Agency on request. Requests to access these datasets should be directed to https://www.folkhalsomyndigheten.se/the-public-health-agency-of-sweden.
