# Supplementary material for: Trust in healthcare and perceived discrimination in Sweden: a fixed effects analysis of individual heterogeneity and discriminatory accuracy
Source: Front Public Health. 2025 Jun 11;13:1557921. doi: 10.3389/fpubh.2025.1557921 (PMC12187740; doi:10.3389/fpubh.2025.1557921)
Supplement: Supplementary file 1 [file Data_Sheet_1.docx]

Supplementary Material

**S1.** Description of the population, and prevalence of low trust in healthcare, with 95% confidence intervals, by categories of age, sex/gender, migrations status, education and perceived discrimination. Those categorized here as having low trust responded *no trust at all* (*very strong*; *fairly strong*; *have no opinion; not that strong* vs. *not at all*). Odds ratios (OR) with 95% confidence intervals (CI) were obtained from regression including the noted variables and adjusting for survey year, using weighted NHPS survey data from 2004–2014.

|  | Population, % | P (95% CI) | OR (95% CI) |
| --- | --- | --- | --- |
| **Trust** |  |  |  |
| High | 97.3% |  |  |
| Low | 2.7% |  |  |
| **Age** |  |  |  |
| 25–34 | 18.1% | 3.6% (3.6–3.7) | 2.06 (2.03–2.10) |
| 35–54 | 41.1% | 3.0% (3.0–3.0) | 1.65 (1.62–1.68) |
| 55–64 | 21.3% | 2.3% (2.3–2.3) | 1.27 (1.25–1.30) |
| >65 | 19.5% | 1.8% (1.8–1.9) | *(Reference)* |
| **Sex/gender** |  |  |  |
| Male | 50.2% | 2.9% (2.9–3.0) | 1.19 (1.18–1.20) |
| Female | 49.8% | 2.5% (2.5–2.6) | *(Reference)* |
| **Migration status** |  |  |  |
| Native-born | 84.1% | 2.2% (2.2–2.2) | *(Reference)* |
| Foreign-born | 15.9% | 5.6% (5.6–5.7) | 2.45 (2.43–2.48) |
| **Education** |  |  |  |
| Low | 64.7% | 3.1% (3.1–3.1) | 1.76 (1.74–1.79) |
| High | 35.3% | 2.1% (2.0–2.1) | *(Reference)* |
| **Discrimination** |  |  |  |
| Yes | 5.0% | 7.7% (7.6–7.9) | 2.78 (2.74–2.82) |
| No | 95.0% | 2.5% (2.5–2.5) | *(Reference)* |

**S2.** Discrimination having occurred in healthcare, using weighted NHPS data from 2004–2005.

| **Discrimination in healthcare** | **n (%)** | **Among those reporting low trust** |
| --- | --- | --- |
| Yes | 223,785 (4.1%) | 146,699 (65.6%) |
| No, but discriminated | 1158,197 (21.5%) | 400,276 (34.6%) |
| No, not discriminated | 4,018,292 (74.4%) | 994,805 (24.8%) |
